# Transient complete atrioventricular block and ST-segment elevation induced by coronary vasospasm due to iatrogenic hyperkalemia: a case report

**DOI:** 10.1186/s13256-020-02644-x

**Published:** 2021-02-11

**Authors:** Miaomiao Cao, Li Chen, Chaofeng Sun, Guoliang Li

**Affiliations:** Department of Cardiovascular Medicine, The First Affiliated Hospital of Xi’an Jiaotong University, 277 Yanta Road, Xi’an, Shaanxi 710061 People’s Republic of China

**Keywords:** Acute coronary syndrome, Hyperkalemia, Coronary vasospasm, Electrocardiogram, Case report

## Abstract

**Background:**

Hyperkalemia and acute coronary syndrome are not only all responsible for syncope related to complete atrioventricular block, but also share parts of electrocardiogram manifestations. Additionally, they influence each other.

**Case presentation:**

A 32-year-old Chinese man presented with severe hypokalemia (1.63 mmol/l) at midnight in the emergency room. He developed unexpected rebound hyperkalemia (7.76 mmol/l) after 18 hours of oral and intravenous potassium chloride supplementation at a concentration of about 10 g/day and a rate of 10 mmol/hour. Subsequently, the patient complained of chest discomfort and dyspnea, followed by syncope for several minutes, approximately 2 hours after potassium reduction treatment had been started. The instant electrocardiogram showed complete atrioventricular block and elevated ST segment in the inferolateral leads, which resolved 15 minutes later, before hyperkalemia was corrected. Combined with mild coronary stenosis and negative myocardial injury markers, transient complete atrioventricular block induced by coronary vasospasm due to iatrogenic hyperkalemia was diagnosed. Normal urine potassium excretion, acid–base state, and other examinations made the diagnosis of hypokalemic periodic paralysis possible.

**Conclusions:**

Hyperkalemia may provoke acute coronary syndrome, and early coronary angiography is an effective strategy for identifying the direct cause of acute complete atrioventricular block.

## Introduction

A number of conditions can cause acute complete atrioventricular block (AVB) and ST-segment elevation. Clinically, acute coronary syndrome (ACS), especially in the case of right coronary artery occlusion, is the most common reason. Hyperkalemia-induced complete AVB and elevated ST segment are also reported but relatively uncommon. Interestingly, hyperkalemia and ACS may coexist; they not only share certain similar electrocardiogram (ECG) manifestations such as ST-segment elevation, T-wave abnormality, or complete AVB as mentioned, but also influence each other. When acute complete AVB and ST-segment elevation occur in individuals with obvious hyperkalemia, the diagnosis of ACS should be considered. Here, we present a case of transient complete AVB and ST-segment elevation induced by coronary vasospasm due to iatrogenic hyperkalemia in a young adult.

## Case presentation

**Fig. 1:**
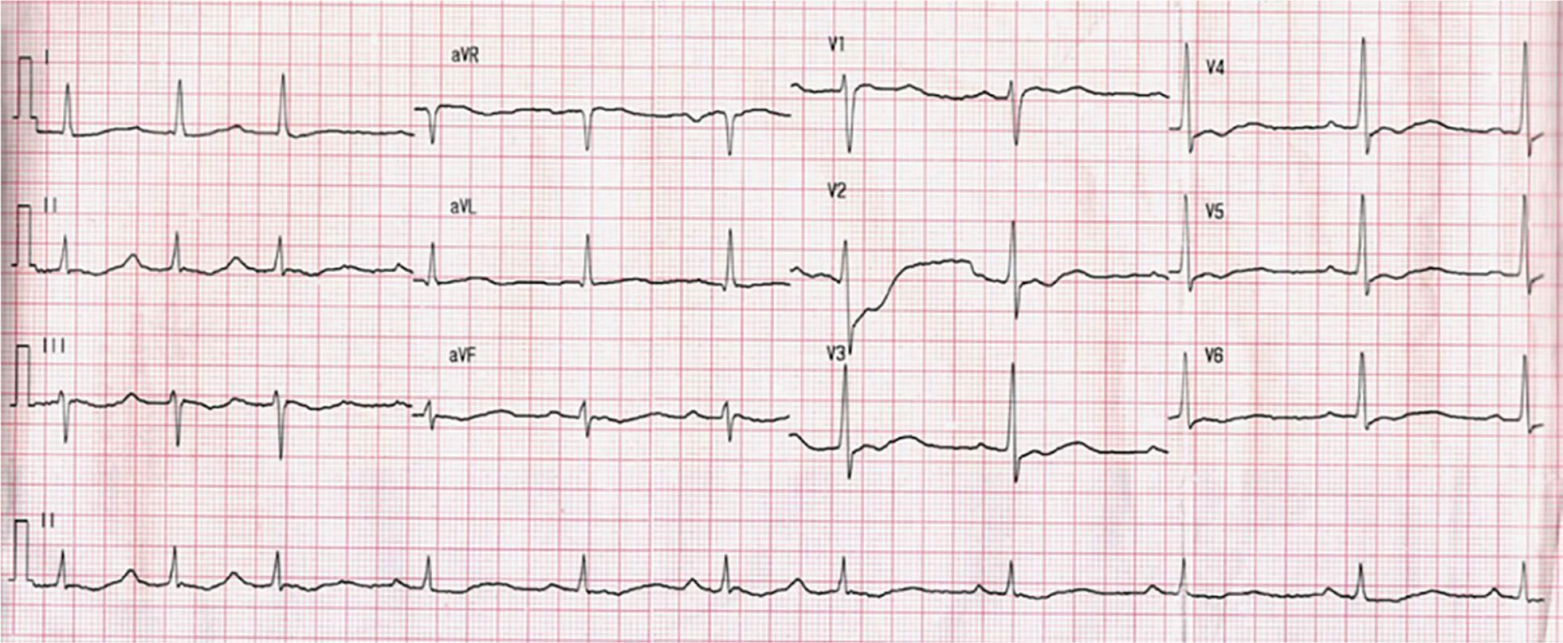
Electrocardiogram on admission revealed decreased T-wave amplitude and prominent U wave with serum potassium of 1.63 mmol/l

A 32-year-old Chinese man presented to the emergency unit at midnight with obvious fatigue and inability to walk by himself. He denied fever, chest discomfort, or shortness of breath. He had a 10-year history of smoking and recurrent weakness of limbs accompanied by hypokalemia over the past 2 years, but no medical records could be accessed. A family history of similar diseases was denied. Physical examination did not provide any remarkable findings, and neurological examination revealed symmetric and decreased muscle strength of the limbs. The ECG on admission revealed decreased T-wave amplitude and prominent U wave (Fig. 1). Initial serum potassium was 1.63 mmol/l (normal range 3.5–5.3 mmol/l). Serum potassium increased to 2.52 mmol/l after 13 hours of oral and intravenous potassium chloride supplementation of about 10 g/day at a rate of 10 mmol/h. Rebound hyperkalemia occurred (7.76 mmol/l) after potassium chloride supplementation by deep vein catheterization for another 5 hours. Treatment including calcium gluconate, insulin and glucose, and diuretic was administered to reduce serum potassium.

**Fig. 2:**
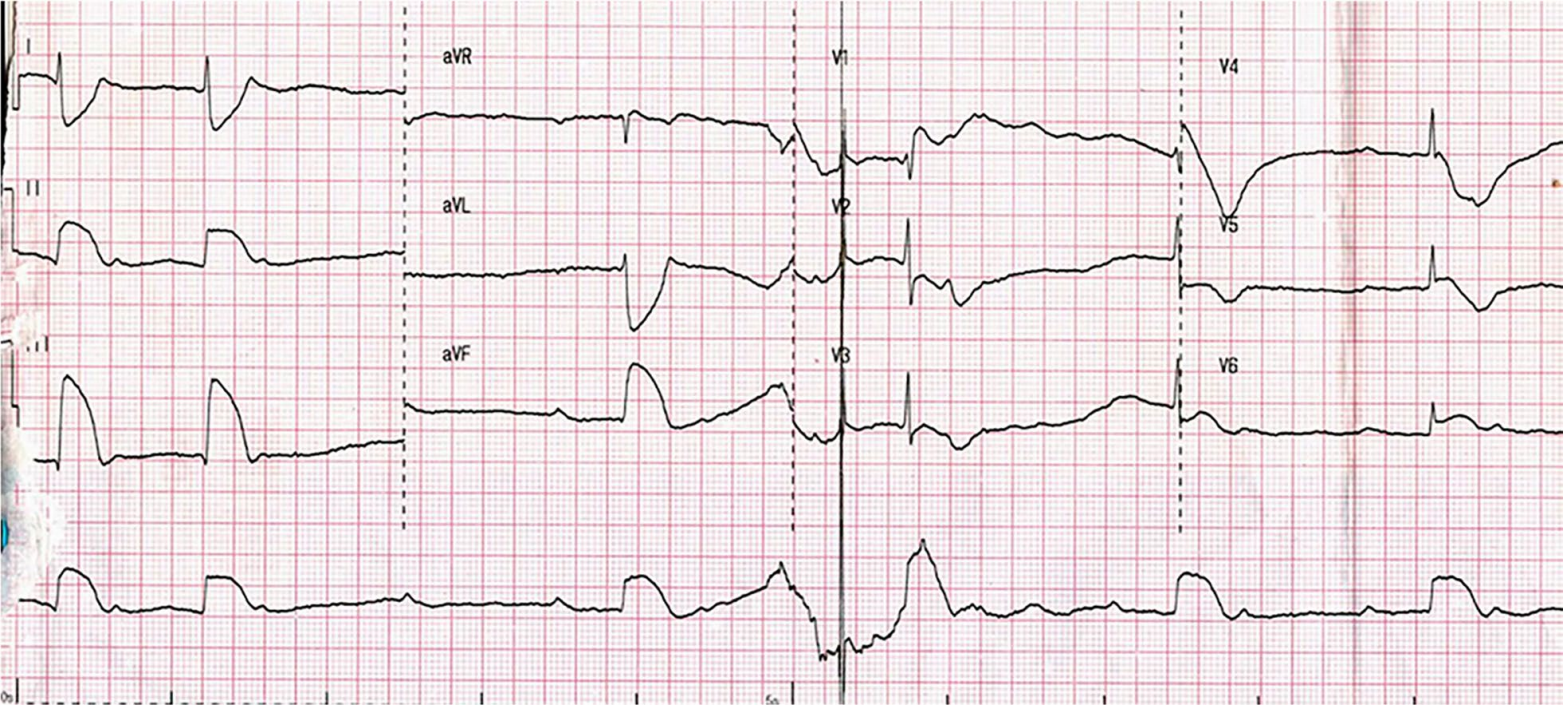
Simultaneous electrocardiogram during syncope exhibited complete atrioventricular block, elevated ST-segment in leads II and III, and atrioventricular block with an instant serum potassium of 7.84 mmol/l

One hour later, the patient complained of gradually aggravated chest discomfort and dyspnea, immediately developing unconsciousness. The simultaneous ECG exhibited complete atrioventricular block (AVB) and elevated ST segment in the inferolateral leads, with instant serum potassium of 7.84 mmol/l (Fig. 2). He was successfully resuscitated after active cardiopulmonary resuscitation but still with dyspnea. Complete AVB and elevated ST segment disappeared 15 minutes later, and the potassium disorder was corrected 10 hours later.

The instant level of high-sensitivity troponin T was  0.024  ng/ml (normal range 0–0.014 ng/ml), creatine kinase (CK) was 185 U/l (normal range 50–310 U/l) and CK-MB was 12 U/l (normal range 0–24 U/l), lactic dehydrogenase (LDH) was 346 U/l (normal range 120–250 U/l), hydroxybutyrate dehydrogenase (HBDH) was 197 U/l (normal range 72–182 U/l), and glutamic oxaloacetic transaminase (GOT) was 71 U/l (normal range 15–40 U/l). Further coronary angiography indicated mild stenosis in the left anterior descending branch and the right coronary artery. Coronary vasospasm was thought to be the direct reason for transient complete AVB and ST-segment elevation. Intravenous diltiazem, oral aspirin, ticagrelor, and high-dose atorvastatin were prescribed. Renal function and arterial blood gas analysis on admission were all normal. Two days after admission, other laboratory tests including thyroid function, renin and aldosterone, cortisol and adrenocorticotropic hormone, glucose and insulin levels, and autoantibodies provided no meaningful clues for hypokalemia. The urinary electrolytes showed normal potassium excretion. Hypokalemic periodic paralysis was considered as the cause of hypokalemia, and genetic testing was subsequently recommended. However, the patient and his family members refused further examinations. One week later, the ECG and serum potassium remained normal, with no symptoms. The patient was eventually discharged on isosorbide mononitrate, diltiazem, aspirin, atorvastatin, and potassium magnesium aspartate to prevent coronary vasospasm and hypokalemia. Quitting smoke was also recommended. At 2-month follow-up, the patient had no chest discomfort or dyspnea, and no obvious abnormalities were found on ECG or 24-hour Holter monitoring.

## Discussion

ST-segment elevation complicating complete atrioventricular block can be found in many clinical settings including ACS [1], myocarditis [2], and hyperkalemia [3]. Clinically, ACS is the most common reason for acute complete AVB and ST-segment elevation. It was reported that complete AVB was found in around 1.9% of patients with ACS and was associated with a higher risk of in-hospital death [1]. There are two possible mechanisms for the generation of acute complete AVB in ACS. One is increased vagal tone in the early stage of ACS, which is sensitive to atropine [4]. Another may be associated with transient ischemia of the atrioventricular conduction system. AVB is most common in acute inferior myocardial infarction because the atrioventricular node blood supply is provided by the atrioventricular node artery, 80–90% of which arises from the right coronary artery [5]. It is believed that immediate angiography is quite helpful for transient AVB and ST-segment elevation with suspected ACS if hemodynamic stability is present.

Hyperkalemia also leads to reversible complete AVB and ST-segment elevation unrelated to myocardial ischemia [6]. The electrophysiological influence of hyperkalemia on myocytes accounts for various ECG abnormalities [7]. Specifically, extracellular high potassium reduces membrane resting potential, causing decreased velocity and amplitude of depolarization. This process eventually slows the conduction in the different regions of the heart, which is reflected on ECG as prolonged P–R interval, absence of P waves, widening of the QRS complex, and even AVB. In addition, hyperkalemia promotes the outflow of intracellular potassium by I_Kr_, shortening the duration of repolarization and action potential. Its uneven effects between epicardial, endocardial, and M cells may result in ST-segment deviation, and sharp, high T waves that resemble ECG manifestations of ACS.

Most interestingly, hyperkalemia and ACS may influence each other. On one hand, the application of certain drugs for ACS, such as angiotensin-converting enzyme inhibitors and potassium-sparing diuretics, may lead to or aggravate hyperkalemia [8]. On the other hand, hyperkalemia can increase the risk of malignant arrhythmia in patients with ACS [9]. Hyperkalemia may also trigger coronary vasospasm and cause complete AVB indirectly. The related mechanisms have not been fully elucidated. Other concomitant electrolyte abnormalities, acidosis, and anoxia under the condition of hyperkalemia may play important roles in stimulating coronary vasospasm [10].

In the present case, transient complete AVB and ST-segment elevation were considered to be induced by coronary vasospasm due to hyperkalemia rather than by hyperkalemia directly. To begin with, complete AVB and ST-segment elevation caused by hyperkalemia can be largely eliminated after serum potassium returns to normal, while resulting from coronary vasospasm is reversed by artery recanalization. Here, transient complete AVB and ST-segment elevation simultaneously disappeared when serum potassium was still at a high level. Then, ST-segment elevation in inferior leads on ECG was consistent with the distribution of compromised coronary artery blood flow, while ST-segment changes were basically consistent between precordial and limb leads for hyperkalemia [11]. Finally, smoking history, unexplained chest discomfort and dyspnea, and transient ST-segment elevation that resolved spontaneously accompanied by mild coronary artery lesions and negative myocardial injury markers supported the diagnosis of coronary vasospasm.

On the other hand, numerous clinical conditions or disorders can cause hypokalemia, such as chronic diarrhea, prescribed drugs, hyperthyroidism, hyperinsulinemia, acid–base disturbance, renal tubular acidosis, Cushing syndrome, Conn syndrome, Gitelman syndrome, and hypokalemic periodic paralysis [12]. To explore the underlying cause of hypokalemia, all information regarding medical history, family history, symptom and physical examination, and simultaneous laboratory tests including serum and urine samples need to be collected at the outset. The present case involved a young man with 2 years of recurrent hypokalemia. Family history and other medical history were denied. A series of laboratory tests revealed no evidence of hyperthyroidism, extrarenal loss or increased renal potassium excretion, abnormal hormone levels, or acid–base disturbance. The diagnosis of hypokalemic periodic paralysis was suspected. As a rare cause of hypokalemia, hypokalemic periodic paralysis is characterized by autosomal dominant inheritance, in which loss-of-function mutations in genes encoding sodium, calcium, and potassium ion channels lead to recurrent hypokalemia and muscle weakness, and may be evoked by a high-carbohydrate diet, strenuous exercise, infection, or other factors. Unfortunately, this patient refused to undergo genetic testing, so the precise evidence for diagnosis was missing. If hypokalemic periodic paralysis is suspected in a patient with hypokalemia, early recognition, more frequent monitoring of serum potassium, and lower dosage may help to prevent rebound hyperkalemia, because these patients may have a spontaneous recovery that results in potassium redistribution.

## Conclusion

Hyperkalemia can directly or indirectly cause malignant arrhythmias. Early coronary angiography might be effective for identifying the direct etiology of acute complete AVB when hyperkalemia and ACS coexist. Prevention and prompt recognition of ACS are always of great importance.

## Data Availability

All data generated or analyzed during this study are original.

## Abbreviations

AVB: Atrioventricular block
ACS: Acute coronary syndrome
ECG: Electrocardiogram
CK: Creatine kinase
LDH: Lactic dehydrogenase
HBDH: Hydroxybutyrate dehydrogenase
GOT: Glutamic oxaloacetic transaminase
